# Rheumatic Fever in Large Cohort of Adolescents in Israel

**DOI:** 10.3389/fmed.2019.00328

**Published:** 2020-01-24

**Authors:** Yossy Machluf, Yoram Chaiter, Rivka Farkash, Anat Sebbag, Daniel Lyon Fink

**Affiliations:** ^1^Medical Corps, Israel Defense Forces (IDF), Haifa, Israel; ^2^Shamir Research Institute, University of Haifa, Haifa, Israel; ^3^Shaare Zedek Medical Center, Jerusalem, Israel; ^4^HaEmek Medical Center, Afula, Israel

**Keywords:** rheumatic fever, prevalence, risk factors, Israel, valvar disease

## Abstract

**Background:** Acute rheumatic fever (ARF) remains a major worldwide healthcare problem, despite its progressive decline in developed countries. The aims of our study were to estimate the prevalence of ARF among adolescents in Israel and to investigate risk factors.

**Methods:** The study population consisted of 113,671 consecutive conscripts 16–19 years old, born during 1971–1993, who completed the medical profiling as part of the recruitment process to the IDF. ARF was diagnosed according to Jones criteria at time of illness, with confirmation relying on medical documentation and cardiologist evaluation including echocardiography.

**Results:** The general prevalence rate of ARF was 0.12%. A downward trend was observed, from 0.19% among those born during 1971–1980 to 0.07% among those born during 1981–1993. Differences in prevalence of ARF were noted in sub-populations based on country of birth and origin—Israel, Ethiopia and the former soviet union (FUSSR). The prevalence rates of valvar disease among ARF+ and ARF– recruits were 15.7% and 0.95%, respectively. In multivariable logistic regression analyses, 4 variables were negatively associated with ARF: later birth year group, female gender, rural residence, youngest child; and 3 were positively associated with ARF: young parents, above normal BMI and valvar disease.

**Conclusion:** Our study provides support for the documented decline in ARF prevalence and describes socio-demographic and anthropometric risk factors including the association with valvar disease and novel risk factors including above normal BMI and young parents, both warranting further investigation which might help in developing societal level prevention strategies.

## Introduction

Acute rheumatic fever (ARF) is a complication of an autoimmune response to pharyngeal infection with Group A β hemolytic *Streptococcus* characterized by rheumatologic, cardiac, and neurologic manifestations, and may lead to chronic morbidity and early death (1–3). ARF is a precursor to rheumatic heart disease (RHD) which may result in irreversible valve damage and heart failure (4, 5). The long-term damage to cardiac valves results from either a single severe episode or from multiple recurrent episodes of ARF (6). Most epidemiologic studies identify RHD rather than ARF, as the diagnosis of ARF is based on clinical findings without a laboratory gold standard (1).

The diagnosis of ARF relies on the Jones criteria, developed in 1944, with subsequent revisions by committees of the American Heart Association (AHA) (7, 8) in 1956 (9), 1965 (10), 1992 (11), reconfirmed in 2000 (12), and most recently modified in 2015 (13). The 1965 and 1992 diagnostic criteria were divided into major and minor. The major criteria for ARF were migratory polyarthritis, carditis, chorea, erythema marginatum, and subcutaneous nodules and the minor criteria were polyarthralgia, fever, elevated erythrocyte sedimentation rate, and/or elevated C-reactive protein and prolonged PR interval on ECG. Diagnosis of ARF is made by the presence of either two major or one major and two minor criteria *plus* evidence of recent streptococcal infection (11). These criteria have been updated recently to relate to the widespread ability to diagnose subclinical endocarditis and the proven need to relax the criteria in moderate and high risk populations (13). A schematic summary of the last version of the Jones criteria is provided by Licciardi et al. (14). According to the recent revision (13), separated diagnostic criteria were set to moderate to high-risk and low-risk populations [incidence cutoff of <2/100,000 school age children (5–14 years old)]. The epidemiological impact of these new guidelines for the diagnosis of ARF was recently shown in a moderate to high risk Italian population, where applying the high risk criteria resulted in a 20.7% increase in the incidence of ARF (14).

The global burden of the disease (GBD) was recently assessed by disease analytic tools and modeling methods, providing an estimation of 33.4 million cases worldwide (15)—more than twice that calculated by means of a systematic review of the literature (16). There is marked heterogeneity in the burden of RHD and lack of accurate data in many countries (15, 17). While the prevalence, incidence, morbidity and mortality burden of ARF and RHD have been decreasing in developed nations since the early 1900's, high rates still persist in socially disadvantaged areas of the world (2, 15). For every clinical case of RHD, there are additional subclinical cases detected by echocardiography (18), and other important complications (19), which were neglected in the GBD study, implying a higher global burden of the disease (20).

A recent meta-analysis of screening studies in low- and middle-income countries (21) showed that the prevalence of clinically silent RHD (21.1/1,000) was almost eight times higher than that of manifest disease (2.7/1,000). RHD prevalence increased with advancing age, from 4.7/1,000 at age 5 years to 21/1,000 at 16 years. Although there is valid criticism of these results (22), it is the best estimate presently available.

ARF rates and trends were also estimated in Israel. A persistent decline in the occurrence of ARF and RHD among the young population from the 1950's until the early 1980's was reported (23, 24). A parallel increase in the prevalence of adult RHD was related to the massive immigration from high prevalence countries (23). Between the years 1996 and 2012, using data from the government statistics center, an average annual incidence of ARF of 2/100,000 was obtained among those under 18 years of age, declining by over 50% during the study period (25). A study using ambulatory clinic records, demonstrated higher annual incidence among the young population (7.5/100,000 school-age children compared to 1/100,000 in those older), males (2.26 times higher), large families, non-Jewish population and rural areas (26). Using two major hospital discharge records in Northern Israel with a predominately Arab population, an annual incidence of ARF was 5/100,000 with a median age of 18 years (27).

Poverty and household overcrowding are associated with a higher risk of ARF (1), as well as insufficient public awareness, health-care services, availability of antibiotic prophylaxis (28), gender and ethnicity (29). In New Zealand, the likelihood of ARF development varied considerably by age, ethnicity, social strata (30), dental caries and sugar intake (31). It is essential for each country to establish its population risk for ARF (32) including within well-defined subpopulations and regions.

The aims of our study were to estimate the prevalence of ARF in a large population of adolescents undergoing recruitment to the IDF. We examined secular trends of ARF during a period of 23 years and its associations with socio-demographic variables, anthropometric indices and valvar disease. We also looked at subgroups of Israeli born recruits compared to those born in Ethiopia and the FUSSR.

## Methods

### Study Population

The Israeli National Military Service Act requires all 17-year-olds to undergo medical profiling at regional recruitment centers. At the end of this process—including history, physical examination, and referral for additional investigation according to findings—a medical profile including appropriate Functional Classifications Codes (FCCs) are assigned to each recruit. FCCs describe the medical status and its severity and are similar to the international classification of diseases coding. No self-reported measurements are accepted. This process is described in detail elsewhere (33, 34).

The computerized database of the northern recruitment center was used for this study. It has been shown to have a stringent, high quality medical process with reliable data (34). The study population consisted of consecutive conscripts 16–19 years old, born during 1971–1993 who completed the medical profiling process during the years 1988–2011 with valid height and weight measures as previously described (35, 36).

### Definitions

History of ARF was reported by the primary care physician and diagnosed according to the relevant Jones criteria. Diagnosis of ARF was made by the presence of either 2 major or 1 major and 2 minor criteria, relying on thorough medical documentation and cardiologist evaluation including echocardiography. Only cases that were identified as meeting the criteria for ARF, by both the cardiologist and the medical committee chairman, were assigned a specific FCC for ARF.

Cardiac lesions were discovered in the recruitment center from either the recruit's personal history, documentation from his/her physician or by the routine and thorough medical examination by two separate clinicians. Any suggestion of cardiac pathology was then investigated by a cardiologist. Cardiac anomalies were all substantiated by echocardiographic study according to accepted criteria. Valvar anomalies included mitral or tricuspid insufficiency or stenosis, aortic or pulmonary insufficiency or stenosis. Valvar anomalies were divided into two groups: non-significant valvar anomalies, and significant valvar anomalies, as described previously (36).

### Statistical Analysis

Characteristics were described by proportions; Univariate analyses included Chi-square or Fisher's exact test to compare categorical variables. A multivariable backward stepwise logistic regression model was conducted to investigate the associations between demographic characteristics, clinical conditions and outcome. Candidate variables for entrance to the model were those which were found to be associated with the outcome in the univariate analysis. The criterion for entrance into the model was a univariate probability value of *P* < 0.05 and *P* > 0.10 for removal from the model. Odds Ratios (OR) and 95% confidence intervals (CI) were calculated. The statistical tests were 2 sided. *P*-value below 0.05 was considered as statistically significant. All analyses were carried out using the SPSS version 24.0 (SPSS, Inc., Chicago, Illinois).

## Results

### Incidence, Prevalence, and Secular Trends

**Figure 1:**
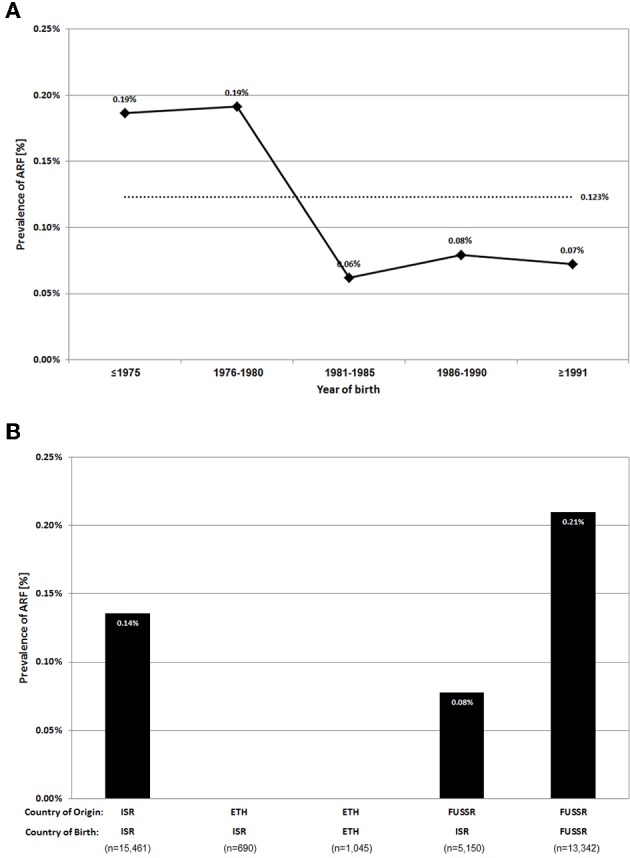
Secular trends of ARF prevalence among whole study population **(A)** (The overall prevalence is indicated by a dashed line) and ARF prevalence among specific sub-populations, stratified by country of origin and country of birth **(B)**.

Our study population included 113,671 recruits aged 16–19 years, born during 1971–1993, and examined during 1988–2011. Of them, 140 recruits had a history of ARF, reflecting a prevalence rate of 0.12%. To investigate secular trends, the study population was grouped into five separate 5-year periods according to birth year (only 2.5 years for the last group, due to sample size considerations). The prevalence among those born during 1971–1980 was 0.19%, ~3 times higher than the prevalence among those born during 1981–1993 (Figure 1A). The deduced mean annual incidence for those born after 1980 was 5.75/100,000 subjects.

As Israel has large immigrant populations, we looked at the subset of Israeli born subjects. Of these 95,527 recruits, 111 had a history of ARF, also reflecting a prevalence rate of 0.12% (Supplementary Figure 1). As the Israeli born subset constitutes the majority of the study population, with similar ARF prevalence rate and trend, we chose to present the detailed findings for the whole population, with differences related to the Israeli born subset when such exist.

The main waves of immigration to Israel during the study period were from the FUSSR and Ethiopia. The study population encompasses both first and second generation immigrants from these countries. ARF prevalence was analyzed among these sub-populations and referenced to Israeli born subjects of Israeli origin (at least third generation Israeli) (Figure 1B). The prevalence rate of ARF among Israeli born subjects of Israeli origin was 0.14%. No cases of ARF were diagnosed among first and second generation Ethiopians. In contrast, the prevalence of ARF was notable among FUSSR origin subjects, higher among those born in the FUSSR (0.21%) compared to those born in Israel to parents from the FUSSR (0.08%).

### Valvar Findings Among ARF Patients

**Figure 2:**
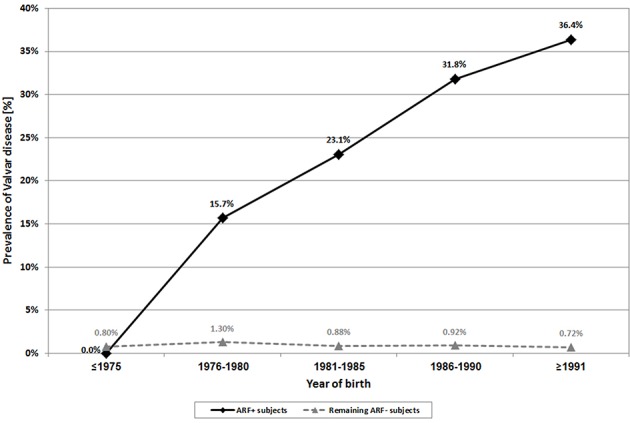
Secular trends of valvar disease prevalence among ARF+ subjects and among the remaining ARF– subjects.

The prevalence of valvar disease among the ARF+ and ARF– populations were 15.7% and 0.95%, respectively. In the ARF+ population there was a significant upward trend (*p* < 0.001) in the prevalence of valvar disease, from none documented among those born in the 1970's up to 36.4% among those born during 1991–1993 (Figure 2). In contrast, the prevalence of valvar disease among ARF– subjects was much lower and relatively stable throughout the study period (0.72%–1.30%).

The prevalence of significant valvar disease among ARF+ subjects was 2.9%, 15 times greater than among ARF– subjects (0.19%). The ratio of significant to all valvar disease was similar in both groups (1:5.5 and 1:5, respectively) [see also (36)].

Similar findings were obtained among subjects of FUSSR origin. The prevalence of valvar disease among the ARF+ population (18.75%) was higher than that among the ARF– population (1%). These rates were similar between subjects of FUSSR origin born in the FUSSR and those born in Israel (data not shown).

### Multivariable Analysis

**Figure 3:**
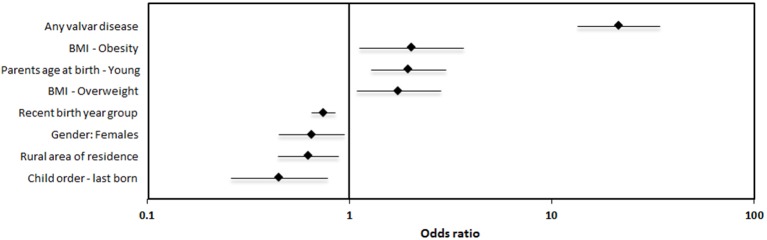
Forest plots of multivariate regression analysis. The correlates [Rhombus – Odds ratio (OR) and bars – 95% confidence intervals (CI)] of variables significantly associated with ARF.

The comprehensive results of the univariate analysis, elucidating associations with ARF are shown in Supplementary Table 1. Those variables significantly associated with ARF were entered into multivariable regression models (Table 1) in a stepwise manner, first including socio-demographic variables (model#1), then adding anthropometric indices (models#2) and finally also valvar disease (model#3). Of the 8 variables included in the comprehensive model#3, 4 were negatively associated with ARF: latter birth year group, female gender, rural residence and being the youngest child; and 3 were positively associated with ARF: having young parents, being overweight or obese and valvar disease (Figure 3). In general, there were no major differences between the 3 models except for a bit stronger and weaker association with “area of residence” and “gender,” respectively (Table 1). Of note, subjects of young parents (combined parents' age at the time of birth) are at higher risk for ARF. Interestingly, both mother's and father's age were independently associated with ARF but mother's age was the dominant association (data not shown).

**Table 1:** Multivariable logistic regression analyses of Acute rheumatic fever (ARF as outcome) and diverse socio-demographic variables, anthropometric indices and valvar disease.

	**Model #1**	**Model #2**	**Model #3**
Variables	**OR** **[95% CI**, ***p*** **value]**	**OR** **[95% CI**, ***p*** **value]**	**OR** **[95% CI**, ***p*****-value]**
**Birth year group**	**0.76** **[0.66–0.86**, ** <0.001]**	**0.74** **[0.65–0.85**, ** <0.001]**	**0.74** **[0.65–0.85**, ** <0.001]**
**Gender**	**0.59** **[0.41–0.85**, **=0.004]**	**0.62** **[0.43–0.91**, **=0.013]**	**0.65** **[0.45–0.94**, **=0.024]**
**Area of residence**	**0.65** **[0.46–0.91**, **=0.013]**	**0.64** **[0.45–0.90**, **=0.010]**	**0.62** **[0.44–0.88**, **=0.007]**
Child order— first born	1.32 [0.64–2.73, =0.449]	1.27 [0.62–2.63, =0.514]	1.20 [0.58–2.48, =0.622]
Child order— last born	**0.48** **[0.28–0.83**, **=0.008]**	**0.47** **[0.27–0.81**, **=0.007]**	**0.45** **[0.26-0.78**, **=0.004]**
Parents age at birth—Young	**1.87** **[1.22–2.86**, **=0.004]**	**1.86** **[1.21–2.84**, **=0.004]**	**1.95** **[1.27–2.98**, **=0.002]**
Parents age at birth—Old	0.77 [0.35–1.69, =0.510]	0.78 [0.35–1.71, =0.530]	0.81 [0.37–1.79, =0.601]
BMI—Under		0.21 [0.03–1.52, =0.123]	0.17 [0.02–1.23, =0.080]
**BMI—Over**		**1.62** **[1.01–2.61**, **=0.045]**	**1.74** **[1.08–2.81**, **=0.022]**
**BMI—Obesity**		**1.86** **[1.03–3.34**, **=0.040]**	**2.02** **[1.12–3.65**, **=0.020]**
Pre-hypertension		1.14 [0.75–1.73, =0.528]	1.13 [0.75–1.72, =0.558]
Hypertension— I & II		1.38 [0.87–2.18, =0.171]	1.31 [0.82–2.07, =0.256]
**Any valvar disease**			**21.37** **[13.41–34.05**, ** <0.001]**
**HOSMER AND LEMESHOW TEST**			
Chi-square	8.828	8.154	9.570
DF	8	8	8
*p*-value	0.357	0.419	0.296

*For each variable the Odds ratio (OR) is shown as well as in brackets []: the 95% Confidence intervals (CI) and p-value. Significance code: all significant differences are marked in bold, and level of significance is color coded: p < 0.05; p < 0.01; and p < 0.0001*.

Although the variable of immigrants vs. Israeli born subjects was not significantly associated with ARF and therefore not included in the multivariable regression models, a noticeable difference between immigrants from different countries—with regard to ARF prevalence—was found (Figure 1B). Inclusion of “country of birth: FUSSR” in the model#3 did not impact the other variables (data not shown) and remained independently significant (OR: 2.01, 95% CI: 1.26–3.21, *p* = 0.003).

## Discussion

### Incidence, Prevalence, and Secular Trends

This is the most comprehensive evaluation to date of prevalence of ARF among the population of northern Israel. We found the prevalence of history of ARF among 16–19 year old recruits stable for those born after 1980 at 0.08%. Since 85% of Israeli cases of ARF were shown to be within the age group of 5–14 years (25), our conservative estimate of prevalence within this age group in the recent period would be 6.6/100,000. We believe there is an advantage in our data as it is based on a detailed medical history from reliable sources but not specifically canvasing for the diagnosis of ARF. Our finding places Israel clearly within the moderate risk countries such that the 2015 updated relaxed Jones criteria should apply (13). The fact that our population is overwhelmingly Jewish with very few Arabs or ultra-religious Jews, both previously shown to have a higher incidence of disease (25–27), establishes our result as of the “lower risk” Israeli population. Applying the new relaxed criteria to all Israeli sub-populations would surely increase the incidence further, as recently shown in Italy (14).

Although ARF is still epidemic in developing countries, our data is consistent with the general downward trend seen in developed countries including Israel. We show a significant decline in ARF prevalence in those born after 1980 and surprisingly stable since.

Israel is a large mix of immigrants and locally born populations. Nevertheless, it is imperative to note that immigrants to Israel came from diverse countries and over the years of our study, mainly the FUSSR and Ethiopia (33, 35), which vary in term of available medical services including diagnosis, monitoring, and treatment. There were no cases of ARF among the Ethiopian origin population of our study. This might be due to a combination of under-diagnosis of those born in Ethiopia and relatively small sample size of those born in Israel. An estimate of RHD among the Israeli-Ethiopian born population has shown that rates are probably in the range of those common in Africa (35). Also, studies from Africa document high rates of RHD and very low rates of ARF (37–40), except in Sudan (41).

Subjects born in the FUSSR were found to have higher rates of ARF (0.21%) than Israeli born subjects, either of FUSSR (0.08%) or Israeli (0.14%) origin. Studies from the Russian Federation from the period of the large wave of immigration have shown a similar high prevalence (0.29%) (29) and in 1994, the incidence of ARF was 18/100,000 children.

### Valvar Findings Among ARF Patients

Our finding of a higher share of valvar lesions among ARF+ patients born after 1980 was previously noted (25) and may be related to the increasing availability of echocardiography and more relevant, the use of color doppler technology, over these years. RHD has recently been convincingly related to inadequate secondary prophylaxis (42), and combined with documented low adherence to secondary prophylaxis (43) in Israel, may explain this finding.

The ratio of significant to all valvar lesions in the ARF+ and ARF– populations is surprisingly similar since ARF has been shown to be a risk factor for more significant lesions (44). This is most probably related to relatively more insignificant lesions discovered in the ARF+ group as they underwent routine echocardiography, irrespective of physical exam findings.

### Multivariable Analysis

Excess of males among ARF patients is consistent with a previous study in Israel (26).

We found a lower risk for ARF in those residing in rural areas and found no association with family size. This is contrary to previous studies that reported higher prevalence of ARF among rural residents and among larger families (1, 26). The typical explanation was that rural areas and larger families meant crowding and lower socio-economic status (SES) resulting in higher carrier rate of Group A β hemolytic *Streptococcus*. Rural residence in northern Israel is not associated with lower SES and overcrowding but rather with a “suburban” middle class, less crowded environment, with availability to high quality medical services—at least comparable to that in urban areas. A similar finding was documented in Bangladesh (45).

This study has uncovered a novel positive association between young parental age and ARF. Younger parental age would be associated with less experience and less awareness of the significance of timely diagnoses and treatment of streptococcal throat infections. This could result in higher rates of ARF. This association implies that parental education might be more effective if directed toward younger families. Similarly, last-born child having a lower risk of ARF as their parents are more experienced and potentially more aware of important child health issues.

Our finding of a positive association between ARF and higher BMI was surprising and not previously described. This association warrants further research.

### Study Strengths and Limitations

The main strengths of our study are the large population size, the long period of data collection where the diagnostic criteria for ARF did not change substantially, a uniform screening process for all subjects including a standardized high-quality and stringent medical process and reliable objective data.

The limitations are that certain populations, such as Arabs and the ultra-orthodox Jews, are under-represented [for further details see Supplementary Materials in (33)]. These populations have been found to have a higher incidence of ARF, making our results conservative but precluding the ability to investigate associations within these populations. Only a third of our population had documented SES according to the Israeli National Bureau of Statistics precluding the inclusion of this important factor in the analyses. Furthermore, the whole population was not screened for RHD and therefore could miss some cases of undiagnosed ARF, most probably in Ethiopian born population. Diagnosis of ARF was made according to Jones criteria, confirmed independently by both cardiologist and medical committee chairman, yielding a specific FCC. Yet, only the FCC was documented in the electronic record. Therefore, the correctness of ARF diagnosis is valid, but the details on the specific criteria related to each case of ARF are missing. Also, the absence of data on recurrences of ARF attacks precluded the analysis of the possible association between severe valvar disease and ARF recurrence. Another issue is the heterogeneity of data collection between the ARF+ and ARF– populations: the ARF+ population would routinely be examined by echocardiography whereas the ARF– population would only be scanned if there was a clinical or other historical diagnosis to suspect heart disease.

## Concluding Remarks

Our study demonstrates a decline in prevalence of ARF among Israeli adolescents (perhaps related to earlier diagnosis and care of strep throat) and—in light of the importance of establishing a country-wide risk of ARF for the new diagnostic parameters (until more recent data are available)—the need for the use of the high risk revised Jones criteria for the Israeli population without exception. It also emphasize existing and novel risk factors—parental young age and above normal BMI—that might help targeting more specific intervention strategies in order to allow early diagnose ARF and prevent cardiac valvar anomalies.

## Data Availability Statement

The datasets generated for this study will not be made publicly available. The IDF Helsinki committee that approved the research has restricted any public approach to the datasets.

## Ethics Statement

This study was approved on the basis of participants' anonymity by an IDF Institutional Review Board ethics committee (Approval number: #1199-2012), and adhered to the tenets of the Declaration of Helsinki. Written informed consent for participation was not required for this study in accordance with the national legislation and the institutional requirements.

## Author Contributions

YM, DF, and YC were involved in all aspects of this study, including, among other aspects: study conception and design, acquisition, analysis, and interpretation of the data. RF and was involved, among other aspects, in study conception and design and data analysis. In addition, all authors were involved in data interpretation and drafting the article, iterative critical revisions for important intellectual content, and final approval of the article. Therefore, each of the authors takes public responsibility for the content, and agrees to be accountable for all aspects of the work including ensuring that questions related to the accuracy or integrity of any part of the work are appropriately investigated and resolved.

### Conflict of Interest

The authors declare that the research was conducted in the absence of any commercial or financial relationships that could be construed as a potential conflict of interest.
