# Supplementary material for: Rheumatic Fever in Large Cohort of Adolescents in Israel
Source: Front Med (Lausanne). 2020 Jan 24;6:328. doi: 10.3389/fmed.2019.00328 (PMC6992586; doi:10.3389/fmed.2019.00328)

Supplementary Materials

# Supplementary Table 1. Univariate analysis of Acute rheumatic fever (ARF) prevalence in population stratified by diverse socio-demographic variables, anthropometric indices and valvar disease, and the derived Risk ratios (RR).

| variable | | Prevalence of ARF within category | RR | Pearson  Chi square P value |
| --- | --- | --- | --- | --- |
|  |  | n=140 |  |  |
| Gender | Female | 0.09% | 0.58 | 0.003 |
|  | Male* | 0.15% |  |  |
| Area of residence | Rural | 0.09% | 0.63 | 0.006 |
|  | Urban* | 0.15% |  |  |
| Number of siblings | 1 | 0.16% | 1.39 | 0.548 |
|  | 2 to 4* | 0.11% | 1 |  |
|  | 5 or more | 0.13% | 1.15 |  |
| Child order | First born | 0.16% | 1.08 | 0.001 |
|  | Other* | 0.15% | 1 |  |
|  | Last born | 0.05% | 0.38 |  |
| Parents age at birth | Young | 0.28% | 2.42 | <0.001 |
|  | Other* | 0.11% | 1 |  |
|  | Old | 0.07% | 0.59 |  |
| Religion | Jewish | 0.12% | 0.76 | 0.225 |
|  | Non-Jewish* | 0.16% |  |  |
| Immigration status | Immigrants | 0.16% | 1.39 | 0.116 |
|  | Israeli born* | 0.12% |  |  |
| Season of birth | Autumn | 0.10% | 1 | 0.566 |
|  | Winter | 0.14% | 1.38 |  |
|  | Spring | 0.13% | 1.29 |  |
|  | Summer | 0.12% | 1.13 |  |
| BMI status | normal* | 0.12% | 1 | 0.014 |
|  | underweight | 0.02% | 0.19 |  |
|  | overweight | 0.18% | 1.55 |  |
|  | obesity | 0.21% | 1.78 |  |
| Hypertension status | normal* | 0.09% | 1 | 0.009 |
|  | pre-hypertension | 0.13% | 1.38 |  |
|  | Hypertension I | 0.20% | 2.06 |  |
|  | Hypertension II | 0.91% | 9.59 |  |
| All valvar diseases | Yes | 2.00% | 19.10 | <0.001 |
|  | No* | 0.10% |  |  |
| Significant valvar diseases | Yes | 1.80% | 15.03 | <0.001 |
|  | No* | 0.12% |  |  |

The reference group within each variable is marked by asterisk (*).

Significance code: all significant differences are marked in bold, and level of significance is color coded: p<0.05; p<0.01; p<0.0001.

**Supplementary Figure 1.** Secular trends of ARF prevalence only among those born in Israel. The overall prevalence is indicated by a dashed line.


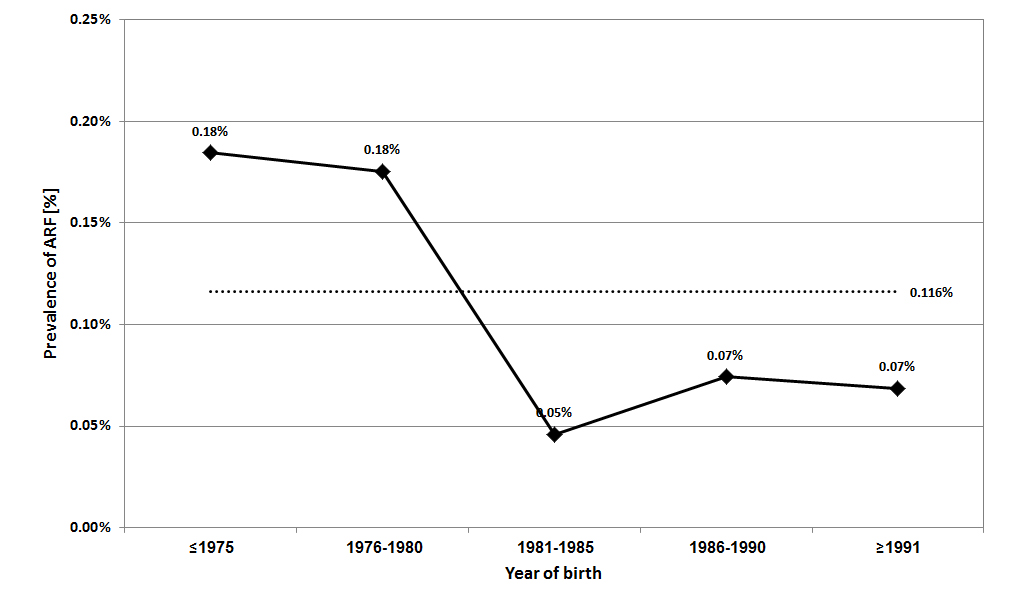

Supplement: Supplementary file 1 [file Data_Sheet_1.docx]
